# Self-reduction of the native TiO_2_ (110) surface during cooling after thermal annealing – *in*-*operando* investigations

**DOI:** 10.1038/s41598-019-48837-3

**Published:** 2019-08-29

**Authors:** M. Rogala, G. Bihlmayer, P. Dabrowski, C. Rodenbücher, D. Wrana, F. Krok, Z. Klusek, K. Szot

**Affiliations:** 1 University of Lodz, Faculty of Physics and Applied Informatics, 90-236 Lodz, Poland; 2 Forschungszentrum Jülich GmbH, Peter Grünberg Institute (PGI-1 & PGI-7), 52425 Jülich, Germany; 3 Forschungszentrum Jülich GmbH, JARA – Fundamentals of Future Information Technologies, 52425 Jülich, Germany; 4 Forschungszentrum Jülich GmbH, Institute of Advanced Simulation (IAS-1), 52425 Jülich, Germany; 5 Forschungszentrum Jülich GmbH, Institute of Energy and Climate Research (IEK-3), 52425 Jülich, Germany; 6 Jagiellonian University, Marian Smoluchowski Institute of Physics, 30-348 Krakow, Poland; 7 University of Silesia, A. Chełkowski Institute of Physics, 40-007 Katowice, Poland

**Keywords:** Surfaces, interfaces and thin films, Chemical physics, Information storage

## Abstract

We investigate the thermal reduction of TiO_2_ in ultra-high vacuum. Contrary to what is usually assumed, we observe that the maximal surface reduction occurs not during the heating, but during the cooling of the sample back to room temperature. We describe the self-reduction, which occurs as a result of differences in the energies of defect formation in the bulk and surface regions. The findings presented are based on X-ray photoelectron spectroscopy carried out *in*-*operando* during the heating and cooling steps. The presented conclusions, concerning the course of redox processes, are especially important when considering oxides for resistive switching and neuromorphic applications and also when describing the mechanisms related to the basics of operation of solid oxide fuel cells.

## Introduction

Titanium dioxide is a model material for a wide group of transition metal oxides, which makes it highly important for understanding the fundamental mechanisms of redox processes present in those materials. These redox processes in titanium dioxide itself can be exploited for numerous applications, which range from catalysis to neuromorphic computing^1–5^. In this work, we present a new element that is the key for correctly modelling the reduction of metal oxides. We extend the existing description of the thermal reduction mechanisms of the TiO_2_ surface by the newly discovered phenomenon of self-reduction, which ultimately determines the spatial distribution of the final stoichiometry.

The fluctuations in stoichiometry of metal oxides define all of their crystallographic and electronic properties and, consequently, their potential applications^6,7^. The initially insulating and chemically inactive TiO_2_ can be transformed into its non-stoichiometric form by the partial removal of oxygen ions. For very small deviations from stoichiometry, the isolated point defects, such as oxygen vacancies and titanium interstitials, are predominant in reduced material^8–11^. However, even point defects with relatively low concentrations have a strong tendency to agglomerate into one-dimensional defect structures^7,12^. When the off-stoichiometry of the TiO_2-x_ crystal is higher, the defects start to arrange into planes^13^, which then causes a shearing process^14^ and finally transforms TiO_2_ into Magnéli phases (Ti_n_O_2n-1_)^15,16^. In real materials, which are initially non-perfect in their form, all of the mentioned features should be taken into account when searching for the mechanisms of any observed and investigated phenomena^17^.

TiO_2_ is considered as a prototype memristive material^17,18^, where resistive switching (RS) phenomena are based on the modifications of local stoichiometry. The redox processes leading to RS are caused by electrical or temperature gradients^19,20^. At the same time the local electrical currents during RS can raise the temperature in the conducting region much above 1000 °C^21^. This process seems to be crucial for the understanding of the details of redox mechanisms. For above reasons we intend to address here the processes responsible for thermal reduction of near surface (near electrode) region of TiO_2_ where RS processes occurred.

**Figure 1:**
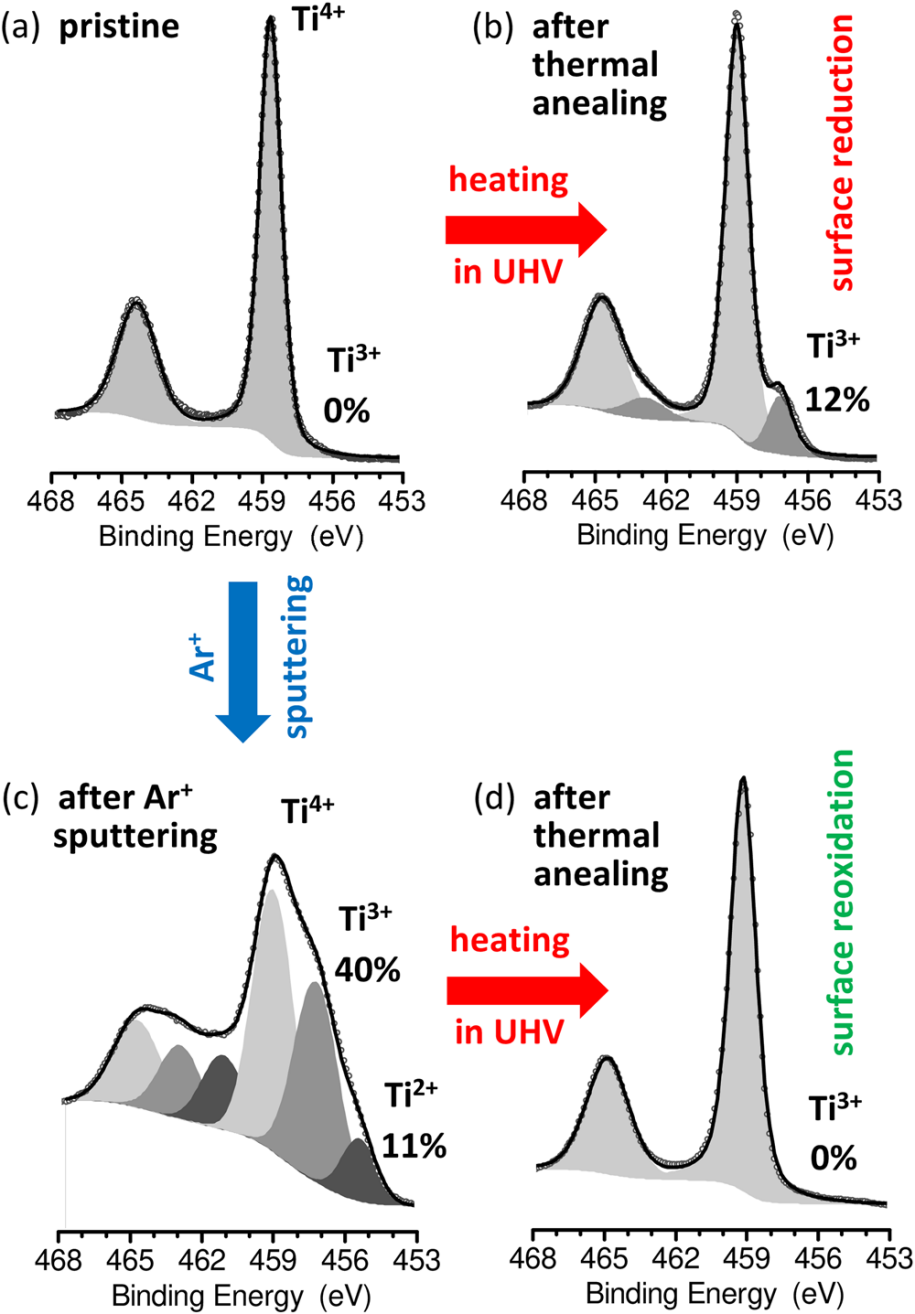
The XPS Ti 2p core line spectra showing the results of thermal annealing of the TiO_2_ crystal in two different cases, 1: when the pristine sample (presented in part **a**) is annealed (1100 °C; 4 h) (presented in part **b**) and 2: when previously Ar + sputtered sample (ion energy 2 kV; 2 min; 10 µA/cm^2^) (presented in part **c**) is annealed (700 °C; 30 min) (**d**). In both cases the thermal annealing caused opposite results in stoichiometry of the surface of material, which is visible as appearance or disappearance of the indication of lower oxidation states (Ti^3+^ or Ti^2+^).

It should be noted, that the reduction of TiO_2_ can be obtained in a variety of ways. Apart from thermal treatment^17^ such process can be forced also by ion sputtering^6,22^, electron irradiation^23^ or electro-degradation^17^. The most common procedure of TiO_2_ reduction is based on the cycling of Ar^+^ sputtering and sample annealing in ultra-high vacuum (UHV) conditions^24^. Such preparation method allows to achieve perfect surface reconstructions, but can complicate the structure of the deeper layers and the bulk. During the Ar^+^ sputtering step a significant disorder is introduced to the material^22^ and only the further step of annealing allows for surface structure restoration. The second common method of oxide reduction, on which we will focus in this work, is thermal annealing of the pristine/stoichiometric material in the oxygen-deficient atmosphere, such as under vacuum conditions^17,25^. The two above-mentioned common methods of preparation are essentially different when considering the effects of thermal annealing processes. The X-ray photoelectron spectroscopy (XPS) measurements allow for observation of the changes in surface stoichiometry (oxidation states of Ti ions) resulting from annealing. Such changes go in fully opposite directions when we consider the two mentioned most common approaches for the metal oxides reduction. When the pristine metal oxide sample is annealed in ultra-high vacuum (UHV) conditions, the thermal treatment is the main factor which results in surface reduction. This is visible for the sample whose spectra are shown in Fig. 1(a,b). Pristine material (Fig. 1(a)) was annealed in UHV at 1100 °C, which led to surface reduction and transition of a part of Ti^4+^ ions to lower (3+) oxidation state (see Fig. 1(b)). This indicates that the thermal annealing process allows for generation of defects in initially pristine material. We are dealing with the opposite situation when applying the reduction process based on cycling of Ar^+^ sputtering and sample annealing in UHV. This method, as shown frequently in the literature, allows for obtaining perfect surface reconstructions^24^, but contains an initial step of Ar^+^ sputtering, which firstly significantly disturbs the native structure of the metal oxide. This is visible in Fig. 1(c) where for a slightly sputtered sample intense changes in the stoichiometry are observed (over 50% of Ti ions have changed their oxidation state). In this method of preparation the thermal annealing step is not intended to reduce (as this is the role of the sputtering), but to re-oxidize the surface. As presented in Fig. 1(d) the initially sputtered sample after thermal annealing in 700 °C completely recovered its stoichiometry. This is possible by the re-segregation of ions during thermal treatment, but it should be noticed that such segregation and surface oxidation takes place in an initially significantly disturbed crystallographic structure. In contrast, in our work we have concentrated on single thermal reduction process, which in its initial step allows for generation of defects in nearly stoichiometric, nearly perfect crystallographic structure, which is especially important i.a. when analysing the resistive switching processes^26^ and their further applications for neuromorphic computing. At the same time this method can give the access to observing the basic phenomena occurring in native (nearly pristine) crystals of metal oxides.

We have shown previously that the thermal reduction can cause more significant changes in the stoichiometry of the surface than of the bulk (visible as differences in phase composition)^17^. However, this analysis was supported by the measurements after cooling the samples down to room temperature. In this paper we investigate the kinetics of defect formation during annealing and cooling. Surprisingly, we prove that the most intense reduction of the surface occurs not at elevated temperatures but during the cooling process and is forced by the influence of the bulk. Such self-reduction in the volume of the crystal is a function of the temperature and can occur as a result of differences in the energies of defect formation in the bulk and in the surface region, which leads to the resegregation of the defects in the material. In contrast to a number of mass transfer processes presented previously for crystals prepared by cycling of Ar^+^ sputtering^11,27^, we describe here the basic phenomena occurring in nearly pristine material. The analyses presented were possible on the basis of the XPS investigation of the chemical states of TiO_2_ surface *in*-*operando* we performed during the heating and cooling processes. This unique approach allows the separation of the processes of oxygen effusion^8^ and the resegregation of defects between the surface layer and crystal interior. With our *in*-*operando* investigations, we detected the phenomena which should be considered when analysing the redox processes in transition metal oxides. Our findings are especially important for the description of basic mechanisms behind resistive switching^20^ and also for optimization of solid oxide fuel cells (SOFCs)^28–31^. As the SOFC commonly operate at temperatures 500–1000 °C, it is crucial to understand the defect migration under such conditions and their evolution upon heating and cooling.

## Results and Discussion

**Figure 2:**
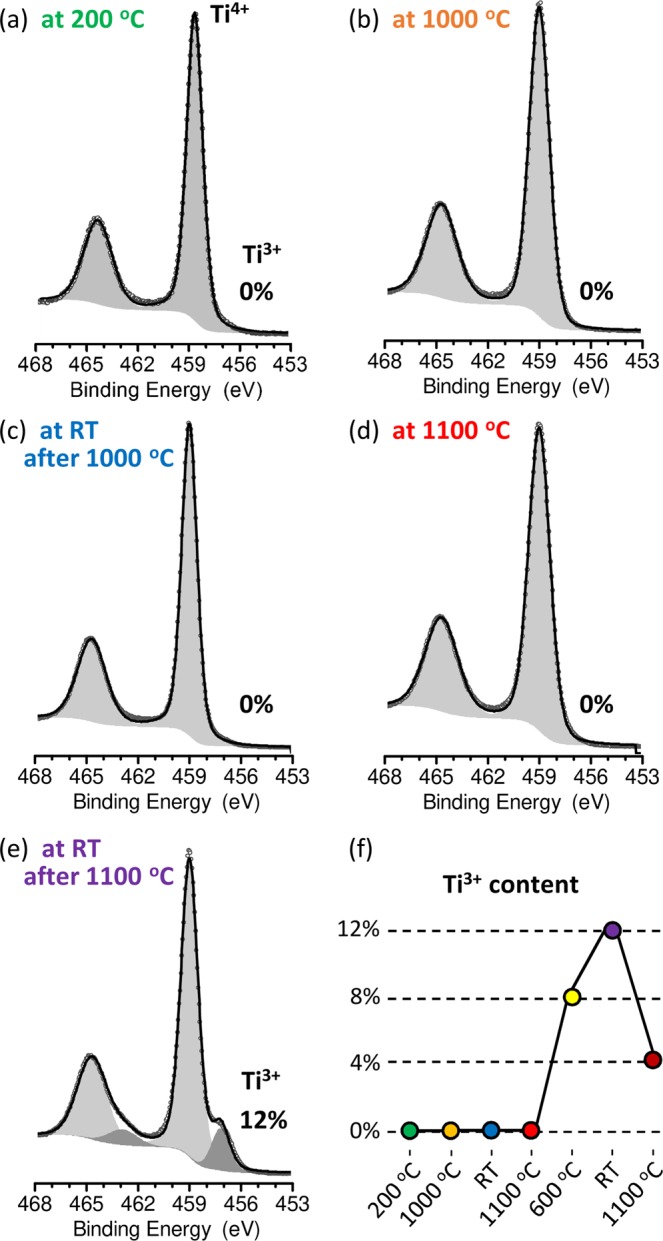
The evolution of Ti 2p XPS spectrum during particular stages of heating and cooling; (**a**) measurements on pristine sample during annealing at 200 °C before the reduction process; (**b**) measurements in 1000 °C (the sample was previously annealed in 700, 800, 900 and 1000 °C for 4 + 4 + 4 + 3 hours); (**c**) at room temperature (after cooling down from 1000 °C, the total time of annealing in 1000 °C was 4 h); (**d**) at 1100 °C (the same sample after additional 3 h annealing in 1100 °C); (**e**) at room temperature (after cooling down from 1100 °C, the total time of annealing in 1100 °C was 4 h); The relative concentrations of Ti^3+^ are marked and also present in plot (**f**).

The pristine rutile TiO_2_ (110) crystal was introduced to the UHV chamber and heated up to 200 °C in order to partially remove the physisorbates (as H_2_O, OH). At the same time, the XPS measurements were performed. The deconvolution of the Ti 2p core line was used to estimate the reduction level of the crystal surface. For the stoichiometric rutile the Ti 2p is a doublet line (2p_1/2_ and 2p_3/2_) containing only the signal from Ti^4+^ states^22^. When surface layer is reduced, the lower oxidation states (3+ or 2+) can be identified as additional doublets shifted to lower binding energies relative to Ti^4+^. The shifts are approximately 1.8 eV and 3.8 eV for 3+ and 2 + states. As is visible in Fig. 2(a), the spectrum for a pristine sample shows only a doublet coming from Ti^4+^ states with the binding energy of Ti 2p_3/2_ line close to 459.0 eV, which indicates correct stoichiometry. A similar spectrum is observed in Fig. 2(b) for the sample during annealing at 1000 °C. In this case, a slight thermal broadening of the core line, proportional to kT is visible, but no evidence of the reduction process is observed by XPS. The effusion of oxygen, which must occur at this temperature^17^ is in our case too weak to be noticed with the changes in the global stoichiometry. This situation persists when the sample is cooled down and the XPS measurement is performed at room temperature (Fig. 2(c)). This indicates that a few hours of reduction in the UHV conditions (see the annealing times and temperatures described in caption of Fig. 2) were not enough to reach the reduction level, which is detectable in XPS measurements with a resolution close to 0.5% (i.e., Ti^3+^/Ti^4+^ < 0.005). However, it should be noticed that at the same time, the sample is slightly reduced, as inferred from its electronic properties^7^. The conditions of thermal reduction described are sufficient for a transformation of TiO_2_ from the insulating to the semiconducting or metallic state^7,17^. This, however, does not necessarily imply a high average concentration of defects, which could be detectable with XPS, but can be related with more local changes in the surface and subsurface layer^26^. Additionally, it should be stressed that, the applied reduction process is thermally driven and should not be compared to the commonly used preparation method when the TiO_2_ sample is in the galvanic contact with the heater and a part of the electric current flows through the crystal supporting the reduction process by the electro-degradation mechanism. Also when performing thermal annealing under the presence of a getter material, such as Si, the oxygen activity can be lowered significantly^32^. With these methods, the noticeable (by XPS) reduction can be achieved for temperatures lower than 1000 °C. In the presented study the influence of the factors which support reduction (as the presence of getter materials in annealed area) is significantly limited. This is related to the construction of the sample holder that allows for high temperature annealing in strictly confined volume, which was a requirement of achieving the possibility of keeping ultra-high vacuum conditions during measurements. Such experimental approach can limit the efficiency of the reduction when compared to previously published results^26^.

As we had not observed a significant reduction after heating up to 1000 °C, we increased the annealing temperature to 1100 °C and measured XPS spectra *in*-*operando*. The result presented in Fig. 2(d) indicates no presence of Ti^3+^ oxidation states on the surface. This shows that again although the oxygen effusion corresponding to the applied annealing condition must introduce some defects^17^, it is not effective enough to be noticed by XPS. The situation changes dramatically after cooling the sample. The spectra measured at room temperature (presented in Fig. 2(e)) shows the appearance of the second doublet with the binding energy of Ti 2p_3/2_ line close to 457.2 eV, which indicate the presence of Ti^3+^ oxidation states. The relative intensities of particular lines prove the reduction process which leads to relatively high concentration of titanium ions in lower oxidation states in the surface layer (approximately 12% of Ti^3+^, which corresponds to Ti/O ratio of 0.53). This indicates that the observed main reduction process of the near surface region of TiO_2_ occurs during the cooling of the sample from high temperature and not during annealing at the maximal temperature, which would be more intuitive. Furthermore, when the sample is heated back up to 1100 °C and kept at this temperature during measurements, the surface reduction level decreases again. The spectra presented in Fig. 2(f) show that further heating caused the decrease of the Ti^3+^ states observed on the surface to about one third. This means that the heating causes the changes in the defects energies, which allow for diffusion of oxygen from the interior of the sample in the direction of the surface, and shows that such diffusion dominates over effusion into vacuum. Furthermore, we have found that the observed reduction process that occurs during the cooling down of the sample from high temperatures is strictly related to the surface reduction. This was checked with an additional experiment in which the surface of the reduced TiO_2_ with the Ti^3+^ concentration, as presented in Fig. 2(e), was removed *in*-*situ* in the XPS UHV chamber with the use of the diamond scraper. In the uncovered the subsurface layers of the crystal no signal of Ti^3+^ oxidation states has been found (see Supplementary Information).

**Figure 3:**
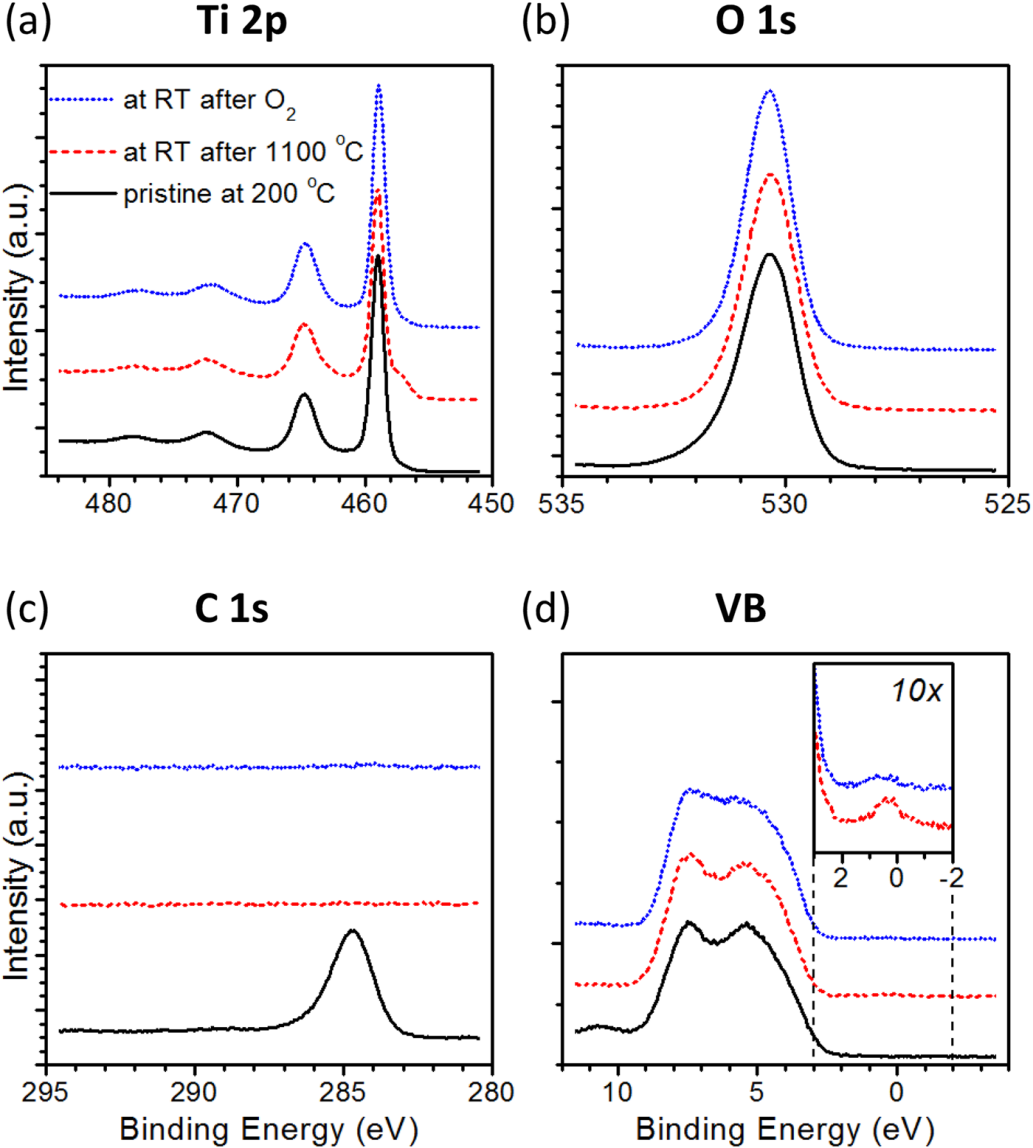
The XPS spectra for the pristine TiO_2_ sample (recorded at 200 °C) – black lines, for the sample after annealing at 1100 °C – red dashed lines, and for the same sample after exposition to 6 L of O_2_ – blue dotted lines. The parts (**a**,**b**) and (**c**) present the titanium, oxygen and carbon core lines, respectively. Part (**d**) shows the valence band spectra, with the inset presenting the magnification of the band gap region, where Ti 3d states can occur.

The stability of the defects generated at the surface of TiO_2_ can be assessed by exposing the crystal to oxidizing conditions. Figure 3 shows the comparison of the XPS spectra after reduction and oxidation of the surface; additionally, the spectrum measured on the pristine TiO_2_ is presented as a reference. The oxidizing process was performed at room temperature, so only transformation of the defects with a high affinity to the oxygen can occur. When comparing the Ti 2p lines (Fig. 3(a)), the decrease of the Ti^3+^ ions concentration after oxidizing is visible. This indicates that the main defects previously presented near the surface can be reoxidized at room temperature and because of that are rather not related with generation of new stable phases in the rutile structure (such as Magnéli phases). Efficient reoxidation at room temperature can occur for point defects (such as oxygen vacancies) or for a relatively unstable cluster of defects such as line defects^26,33^. Hence such defects should be consider as mainly present in the investigated sample. However, as after oxidation the concentration of Ti^3+^ ions does not vanish completely, but remains at the level of 2%, we can assume that also some more stable nonstoichiometric clusters were in the minority generated during the reduction process. The changes observed in the Ti 2p line are accompanied by the changes visible in the valence band structure, as presented in Fig. 3(d). The distinct Ti 3d state present in the band gap of the crystal after reduction is significantly lower after oxidation, which indicates that the electrical conductivity of the reduced TiO_2_ surface is highly influenced by the presence of oxygen in the crystal surroundings. Taking into account that the reoxidation process takes place at room temperature, we can assume that the main defects in the surface layer are oxygen vacancies. The reoxidation of titanium interstitials would require Ti ions migration^10^ and will be ineffective in room temperature^8^. The spectra presented in Fig. 3 additionally indicate the lack of carbon compounds on the TiO_2_ surface after the reduction process. This is visible in both the vanishing of the C 1 s signal (Fig. 3(c)) and the increasing of the symmetry of the O 1 s line (Fig. 3(b)) after reduction. The carbon-based contamination of the TiO_2_ surface is intended to desorb during annealing of the crystal above 700 °C^34^, but what is important for the described processes is that it also does not reappear on the surface during cooling down to room temperature, when the reduction of the surface occurs. Furthermore, carbon does not play any role during the observed reoxidation. Additionally comparing the Ti 2p and O 1 s line after annealing confirms the pure chemical character of splitting the Ti 2p line during reduction (for 4+ and 3+) and excludes the charging effect. If the discussed changes came from charging effect, they would also be visible on the O1s line (which in our case is unaffected).

**Figure 4:**
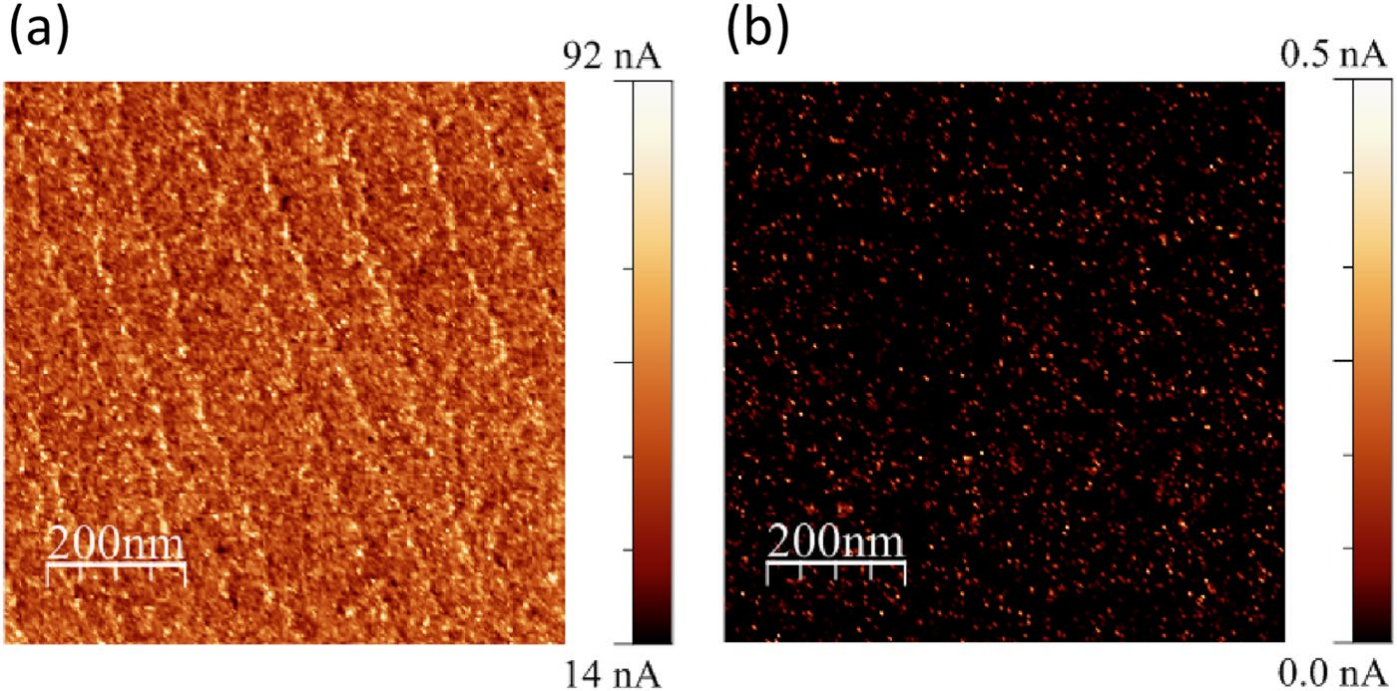
The LC-AFM map of local conductivity of the TiO_2_ surface (recorded at room temperature) for: (**a**) the sample after annealing at 1100 °C; and (**b**) for the same sample after exposition to 6 L of O_2_. The polarization between the AFM probe and surface was 100 mV.

The conductivity of the surface that was reduced during cooling down from 1100 °C is relatively high, which is visible in the LC-AFM^35^ results presented in Fig. 4(a) and confirmed by the observation of valence band spectra (see Supplementary Information). However, the defects responsible for the presence of the electrical conductivity of the surface layer are easily oxidized when the surface of the reduced crystal is exposed to oxidizing conditions^36^. The LC-AFM map of the sample after having been oxidized at room temperature is presented in Fig. 4(b), showing the dramatic decrease in the local conductivity. During oxidation, the surface is easily transformed back into the insulating state at some points, but non-zero conductivity spots, which are probably the exits of the network of conducting firmaments, remain visible^26,34^. This is accompanied by the decrease in the concentration of Ti^3+^ ions in the near surface region, as already discussed.

**Figure 5:**
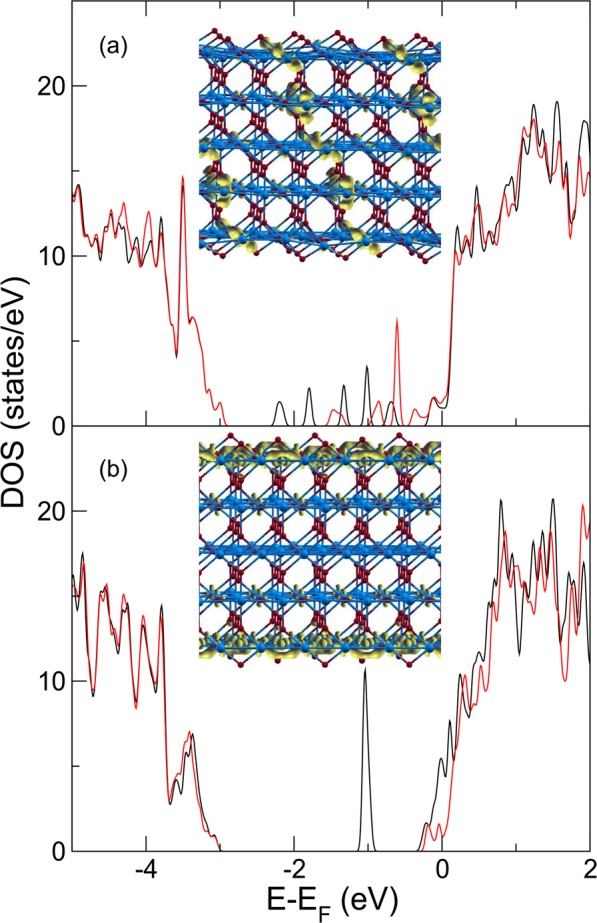
(**a**) Density of states (DOS) of a (110) oriented rutile TiO_2_ film with six oxygen vacancies in a quasi-linear arrangement, threading the film. A metallic band appears in the bulk band gap and is quantized due to the finite film thickness into 6 peaks. Black and red lines show the spin-up and spin-down DOS, respectively. An isosurface of the charge density associated with the in-gap states is shown in yellow in the inset. Blue and red atoms symbolize Ti and O, respectively. The in-plane unit cell is 3 × 2 and the DOS is given per in-plane unit cell. (**b**) The DOS of the same film with vacancies now confined to the upper and lower surface (one vacancy per two in-plane unit cells). The charge density of the shallow states at the conduction band is shown in the inset. The colour code is the same as in panel (a).

As presented in these experiments, the significant surface reduction occurred during cooling, a fact that should be explained by the internal ion transfer between the surface and bulk. To rationalize these findings, we look at the energetics of oxygen defects in TiO_2_ in bulk and at the (110) surface. Vacancy formation energies have been calculated using density functional theory (DFT), with different authors finding that at the surface, this energy can be about 0.8 eV lower than in the bulk^37–41^. Of course this value is just an inner energy while formation enthalpy will include an entropic contribution, both from the configurational and vibrational entropy. To estimate the latter in a crude fashion, we can follow the model of Vineyard and Dienes^42^ and realize that the number of broken bonds at the surface is one less than in the bulk and assume that the vibrational frequency of a broken bond is reduced by a factor of 2. At this limit, we expect to gain 0.3 kT per vacancy at the surface, i.e., about 35 meV at 1100 °C. Although the estimation is crude, it is doubtful that vibrational entropy will be sufficient to overcome the vacancy formation energy difference mentioned above. If we want to determine a layer-dependent vacancy concentration profile that depends on a layer-dependent vacancy formation enthalpy, the vacancy density at the surface is given by:

1 $${c}_{surf}={c}_{bulk}\cdot {e}^{\frac{\Delta E-kT\Delta {S}_{v}}{kT}}$$

where ΔE is the vacancy formation energy difference between the bulk and surface, while ΔS_v_ is the corresponding difference in the vibrational entropy (more details are presented in Supplementary Information). At the limit of high defect densities, it is necessary to consider two effects, namely: the high concentration of defects can lead to a clustering of oxygen vacancies, both in bulk and at the surface. This will considerably modify the vacancy formation energies, e.g., the linear arrangements of vacancies were predicted to have 1.1 eV lower formation energies than those isolated in the bulk^43^, while on the surface, DFT calculations predicted that a divacancy is 0.25 eV more stable than two isolated oxygen vacancies^41^. Since these energies sum up to a small value and they were obtained using different computational models, we simulated two setups of bulk and surface clustered defects in a (110) oriented 5 layer TiO_2_ film with an in-plane 3 × 2 unit cell, resulting in 174 atoms and 6 vacancies that were clustered linearly in the bulk or confined to the surface (Fig. 5 insets of panels (a,b)). We used DFT + U method as described by Park *et al*.^44^. The values for U_p_ and U_d_ were 6 and 8 eV, respectively, to obtain a good description of the bulk band gap (2.7 eV). The full-potential linearized augmented plane wave method in thin film geometry^45^ as implemented in the FLEUR code^46^ was employed with a product muffin-tin radii time plane wave cut-off of 7.1 and a sampling of the reciprocal space with a 2 × 3 k-mesh. All atomic positions were allowed to relax until the forces did not exceed 60 meV/A. The density of the states shows that the former case gives rise to a broad conduction band through the film, which is quantized due to the finite film thickness (Fig. 5(a)), while the latter exhibits shallow states at the conduction band and a spin-polarized, in-gap state (Fig. 5(b)). As per oxygen vacancy, it turns out that the arrangement at the surface is now 57 meV more favourable than the filament threading the film. Keeping in mind that these are only two favourable arrangements out of a few that were investigated and, in reality, that a statistical distribution over many more configurations will be present, we insert this energy difference into Equation (1). At 1100 °C, the ratio of surface to bulk vacancies is found to be about 1.2, while cooling to room temperature increases this ratio to 6.7, suggesting a substantial increase of vacancies at the surface due to cooling. As magnetic order also plays a dominant role in the stabilization of the surface vacancy ordering^41^, the loss of magnetic ordering at higher temperatures might also lead to a destabilization of surface vacancies at high temperatures. Despite the uncertainties of the theoretical model when used to describe a situation far from equilibrium, we estimate that the energetics of bulk- and surface vacancies can be comparable within a range that corresponds to the temperature variations occurring during the reduction process. Such a case permits the observation of a highly efficient self-reduction phenomenon, wherein an initially slightly reduced material from the internal processes leads to the formation of a significantly reduced thin layer in the surface region. In such a model, the surface reduction is not an independent process described only by the temperature and partial oxygen pressure, but is generally a bulk-assisted phenomenon that leads to the resegregation of defects. The observed cooling-induced resegregation should, however, mainly occur in the temperature regime that provides conditions for the migration of defects^7^. In the case presented, the main reduction occurs during cooling down of the sample, but when the temperature was still above 500 °C. The concentration of Ti^3+^ oxidation states measured at 600 °C was at the level of 8% (see Supplementary Information), while it increased to 12% after cooling down to room temperature.

## Conclusions

In conclusion, we found that the thermal reduction observed for the TiO_2_ surfaces annealed in UHV conditions is, in fact, the result of a bulk-assisted process, when the crystallographic defects migrate from the bulk to the surface during cooling of the sample crystal. Our theoretical analyses are strongly supported by *in*-*operando* XPS measurements during annealing of the TiO_2_ crystal. As we observed at elevated temperatures, the removal of oxygen from the material occurs, but the concentration of defects in the surface layer is relatively low. This changes during the cooling down process, when the location of the defects on the surface becomes significantly more favourable than in the bulk. During cooling, the oxygen is transferred in the direction of the bulk in order to minimize the energy of the system, which leads to self-reduction of the surface layer. The described redox mechanism can play a significant role in the formation of non-stoichiometric Ti-O systems. As TiO_2_ is a model oxide system, the findings presented should be taken into account when studying the basic phenomena behind transition metal oxide-based applications, especially in those operating at high temperatures, such as SOFCs. The self-reduction during cooling should be also carefully considered when analysing the behaviour of memristive/neuromorphic devices in which current flow may lead to rise of local temperatures.

### Experimental methods

In our investigations we used rutile TiO_2_ single crystal with epitaxy-ready (110) surface. The crystal was annealed *in*-*situ* in the UHV chamber of the XPS setup. For the XPS investigations, the monochromatic Al K-alpha source was used, the XPS sampling area had the diameter close to 1 mm and the sample-detector angle was set to 45°. The customized resistive heating stage with a low power heating element allowed for performance of the XPS measurements during annealing (*in*-*operando*). The pressure level in the chamber during annealing of the sample at 1100 °C was kept below 7·10^−9^ mbar, which facilitates constant XPS probing. The 5 × 5 × 0.5 mm^3^ TiO_2_ crystal was mounted on a ceramic heater (pyrolytic graphite composite element covered by pyrolytic boron nitride) by clamping block. The sample area available for measurements was 3 × 3 mm^2^. Both heater and clamping block were separated from the sample with platinum foil and the grounding of the sample was provided by a platinum electrode. The heating temperature was measured on the clamping block near the surface of the sample by S type thermocouple (platinum rhodium/ platinum), which provided the accuracy of measurements of sample temperature better than 35 °C (confirmed also by pyrometric method). The charging compensation for unreduced samples during XPS measurement was ensured by the electron gun, however for temperatures above 300 °C we observe no charging effect (probably because of the slight reduction of material) and the charge compensation was not needed. The UHV chamber was equipped with a diamond scraper tool, allowing for the *in*-*situ* removal of the surface layer. The UHV chamber was also equipped with a system allowing for dosing of high purity (99.9999%) O_2_ gas and Ar^+^ ion sputtering gun. A similar heating stage and dosing system were mounted in the atomic force microscopy (AFM) chamber. The AFM setup allowed for high resolution local conductivity measurements (LC-AFM)^35^ with the use of a Pt-coated AFM tip.

## Supplementary information

Supplementary Information

## Data Availability

All data generated or analysed during this study are included in this published article (and its Supplementary Information files).
