# Supplementary material for: Self-reduction of the native TiO2 (110) surface during cooling after thermal annealing – in-operando investigations
Source: Sci Rep. 2019 Aug 29;9:12563. doi: 10.1038/s41598-019-48837-3 (PMC6715630; doi:10.1038/s41598-019-48837-3)
Supplement: Supplementary file 1 — Supplementary Information [file 41598_2019_48837_MOESM1_ESM.pdf]

Supplementary Information

Self-reduction of the native TiO_2 (110) surface during cooling after thermal annealing – in-operando investigations

M. Rogala,* [a,b] G. Bihlmayer,[b,c,d] P. Dabrowski,[a] C. Rodenbücher,[e] D. Wrana,[f] F. Krok,[f] Z. Klusek [a] and K. Szot [b,c,g]

[a] University of Lodz, Faculty of Physics and Applied Informatics, 90-236 Lodz, Poland
[b] Forschungszentrum Jülich GmbH, Peter Grünberg Institute (PGI-1 & PGI-7), 52425 Jülich, Germany
[c] Forschungszentrum Jülich GmbH, JARA – Fundamentals of Future Information Technologies, 52425 Jülich, Germany
[d] Forschungszentrum Jülich GmbH, Institute of Advanced Simulation (IAS-1), 52425 Jülich, Germany
[e] Forschungszentrum Jülich GmbH, Institute of Energy and Climate Research (IEK-3), 52425 Jülich, Germany
[f] Jagiellonian University, Marian Smoluchowski Institute of Physics, 30-348 Krakow, Poland
[g] University of Silesia, A. Chełkowski Institute of Physics, 40-007 Katowice, Poland

** e-mail: rogala@uni.lodz.pl*

SUPPLEMENTARY X-RAY PHOTOELECTRON SPECTROSCOPY DATA FOR THE SAMPLES PREVIOUSLY ANEALED AT 1100 °C.

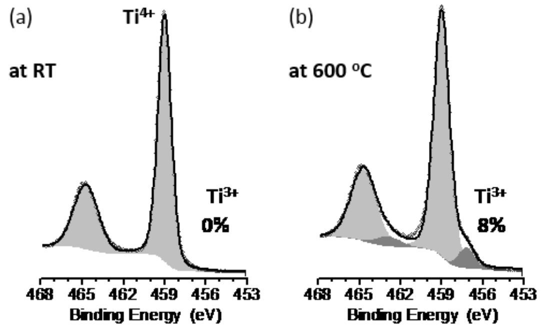

Figure S1. The XPS Ti 2p core line spectrum of TiO_2 (110) surface; (a) after annealing at 1100 °C and removing the surface layer with the use of the diamond scraper; (b) during cooling down after annealing in 1100 °C when the temperature level reaches 600 °C.

The XPS investigations proved the surface localization of the intense reduction process. The surface of the reduced TiO_2 with the Ti^{3+} concentration, as presented in Figure 2(e) in the main text, was removed with the use of the diamond scraper. Such a process performed *in-situ* in an XPS UHV chamber uncovered the subsurface layers of the crystal, which indicated no presence of Ti^{3+} oxidation states, as presented in Figure S1(a).

The TiO_2 (110) surface is mainly reduced during cooling of the sample down from 1100 °C, when the temperature is still above 500 °C. This is visible when analysing the concentration of Ti^{3+} oxidation states at particular temperatures and is presented in Figure S1(b), where XPS data measured at 600 °C are shown. At this temperature, the concentration of Ti^{3+} states was at a level of 8%, while it only increased to 12% after cooling down to room temperature.

THERMODYNAMIC MODEL INCLUDING CONFIGURATIONAL ENTROPY

In the manuscript we derived a relationship between the surface and bulk concentration of vacancies, neglecting the configurational entropy. Then, considering the exchange of vacancies between the surface and bulk layers, S ↔ B_1 ↔ B_2 ↔ …, the rate equations can be written:

$$\frac{\partial[S]}{\partial t} = -k_1[S] + k_2[B_1];\ \frac{\partial[B_1]}{\partial t} = -2k_2[B_1] + k_1[S] + k_2[B_2];$$

$$\frac{\partial[B_n]}{\partial t} = -2k_2[B_n] + k_2[B_{n-1}] + k_2[B_{n+1}]\ \forall\, n \geq 2.$$

In the steady state limit, it follows that:

$$[S] = \frac{k_2}{k_1}[B_1] = e^{\Delta H/kT}[B_1];\ \ [B_n] = [B_{n-1}]\ \forall\, n \geq 2$$

resulting, with ΔH=ΔE-$T\Delta S^v$, in equation (1) in the main text.

To include vibrational entropy, we consider a TiO_2 film consisting of 24 layers and an overall concentration of oxygen vacancies of 0.5%, where the topmost layer will be denoted as the surface. The difference between the vacancy formation energies on the surface and bulk is again ΔE = E_b-E_s > 0. As discussed in the main text, the vibrational entropy at the surface is assumed to be 0.3 kT lower than in the bulk. A layer with defect concentration c will have a configurational entropy of $S^{cf}(c) = k\left[\ln(c) + (1-c)\ln(\frac{1}{c} - 1)\right]$. All bulk layers have the concentration c_B, while the surface layer has c_s = 0.12 – 23 c_b. The free enthalpy of the system can be written as:

$$H = 0.12(E_s - TS_s^v) + 23c_b(\Delta E - T\Delta S^v) - (0.12 - 23c_b)TS^{cf}(0.12 - 23c_b) - 23c_bTS^{cf}(c_b).$$

This expression can be minimized with respect to c_b for a given temperature T to find $c_b(T)$. Combining the last two terms of this equation in $\Sigma^{cf}(c_b)$, we can derive these values from the equation:

$$\frac{\Delta E}{kT} = \frac{1}{23}\frac{\partial \Sigma^{cf}(c_b)}{\partial c_b} + \frac{\Delta S^v}{kT} \qquad \text{(S1)}$$

displayed in Figure S2.

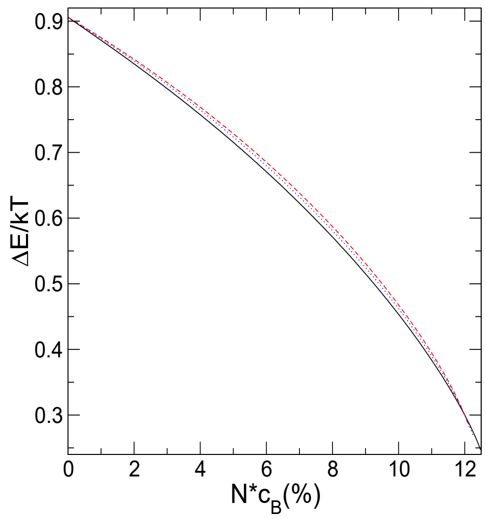

Figure S2. The black line illustrates eq. (S1) for ΔS^v=0.3kT. If $\Delta E/kT$ > 0.9, the bulk vacancy concentration tends to zero and all vacancies gather at the surface layer. For $\Delta E/kT$ < 0.3, the surface layer gets depleted with respect to the bulk – an effect of the vibrational entropy difference. Note that the number of layers, N, is 24. Due to the boundary conditions, c_b cannot exceed 0.52%. The blue (dotted) and the red (dashed) lines are computed for N=48 and N=100 layers, respectively.

We see that for ΔE = 0.8eV it is not possible to stabilize any vacancies in the bulk if kT < 0.88eV. This corresponds to an unreasonably high temperature of 10200K. Assuming ΔE = 57 meV and T=1100 °C, the bulk concentration is about 0.4% and, at the surface, c_s=2.8%. Lowering the temperature to 500 °C is already sufficient to stabilize 10.5% of the vacancies at the surface layer, while at RT all vacancies are located at the surface.

Of course, the model presented here is fairly crude, both in the treatment of vibrational entropy (assuming the Vineyard-Dienes model), as well as in the configurational entropy (using the point defect limit). However, we must see that ΔS^v merely vertically shifts the curve in Figure S2 and adds or subtracts a linear term in $\Delta E/kT$. In reality, we also assume a narrowing in the configuration space in both the surface and bulk layers, corresponding to a rescaling of the derivative of Σ^{cf} and lowering of c_b for a given temperature. We assumed that during the heating and cooling cycles, the topmost 24 layers are accessible to the newly created vacancies. It should be noted though that this number is merely illustrative and the model can be generalized to N layers with the same total number of vacancies in the film. As is shown in Figure S2, the general features are not significantly affected if we scale the bulk concentration with the number of layers.

References:

[1] C. Rodenbücher, G. Bihlmayer, W. Speier, J. Kubacki, M. Wojtyniak, M. Rogala, D. Wrana, F. Krok, and K. Szot, Nanoscale **10**, 11498 (2018).
